# A new species of *Pancorius* Simon, 1902 (Araneae, Salticidae) from Guizhou, China

**DOI:** 10.3897/BDJ.10.e87620

**Published:** 2022-07-07

**Authors:** Jiahui Gan, Xiaoqi Mi, Cheng Wang

**Affiliations:** 1 Guizhou Provincial Key Laboratory for Biodiversity Conservation and Utilization in the Fanjing Mountain Region, Tongren University, Tongren, China Guizhou Provincial Key Laboratory for Biodiversity Conservation and Utilization in the Fanjing Mountain Region, Tongren University Tongren China

**Keywords:** East Asia, jumping spider, morphology, taxonomy

## Abstract

**Background:**

The jumping spider genus *Pancorius* Simon, 1902 is represented by 40 species primarily distributed in East, South and Southeast Asia. Amongst these, 10 (including eight endemics) are known from China.

**New information:**

A new species, *Pancoriuslui* **sp. nov.**, is diagnosed and described, based on both sexes from Yuntai Mountain in Guizhou of China. Diagnostic photos and a distribution map are provided.

## Introduction

The genus *Pancorius* Simon, 1902, a member of the speciose subtribe Plexippina Simon, 1901, currently contains 40 large, stocky species restricted to Asia (except for *P.crassipes* (Karsch, 1881) which was introducted to Poland), with their highest diversity in East and South Asia (Maddison 2015, World Spider Catalog 2022, Metzner 2022). Although the number of known species has considerably increased thanks to a series of recent studies (Caleb et al. 2019, Wang and Wang 2020, Kanesharatnam and Benjamin 2021, Logunov 2021), the genus remains poorly known due to the lack of a global revision. Furthermore, more than half (23) of the species are currently known only from a single sex and seven species cannot be identified due to the lack of diagnostic illustrations. To date, 10 species have been recorded from China, of which eight are endemics and six are known only from a single sex (Wang and Wang 2020, World Spider Catalog 2022).

In our recent study on the jumping spiders of Yunnan-Guizhou Plateau, a *Pancorius* species was recognised as new to science which is described herein.

## Materials and methods

All specimens were collected by beating shrubs and deposited in the Museum of Tongren University, China (TRU). The specimens were examined with an Olympus SZX10 stereomicroscope. After dissection, the epigyne was cleared in trypsin enzyme solution before examination and imaging. The left male palp was used for the descriptions and illustrations. Photos of the copulatory organs and habitus were taken with a Kuy Nice CCD mounted on an Olympus BX51 compound microscope. Compound focus images were generated using Helicon Focus v. 6.7.1.

All measurements are given in millimetres. Leg measurements are given as: total length (femur, patella + tibia, metatarsus, tarsus). The abbreviations used in the text and figures are as follow:

**AERW** anterior eye row width; **AME** anterior median eye; **ALE** anterior lateral eye; **AS** anterior chamber of spermatheca; **CD** copulatory duct; **CO** copulatory opening; **E** embolus; **EF** embolic flange; **EFL** eye field length; **FD** fertilisation duct; **MS** median septum; **PERW** posterior eye row width; **PL** posterior lobe; **PLE** posterior lateral eye; **PS** posterior chamber of spermatheca; **RTA** retrolateral tibial apophysis; **SD** sperm duct.

## Taxon treatments

### Pancorius lui

Gan, Mi & Wang, 2022 sp. n.

BB7E8A32-96D5-5DEB-9A62-6CBFB3BBF639

5DC16EFA-8D11-4DD1-9BFB-BA8E52DB4539

#### Materials

- **Type status:** Holotype. **Occurrence:** individualCount: 1; sex: male; **Taxon:** scientificName: *Pancoriuslui* sp. nov.; order: Araneae; family: Salticidae; genus: Pancorius; **Location:** continent: Asian; country: China; countryCode: CHN; stateProvince: Guizhou; county: Shibing; locality: Yuntaishan Scenic Area; verbatimElevation: 900-1000 m; decimalLatitude: 27.13367; decimalLongitude: 108.10883; **Identification:** identifiedBy: Cheng Wang; **Event:** year: 2015; month: 7; day: 30
- **Type status:** Paratype. **Occurrence:** individualCount: 11; sex: 7 males, 4 females; **Taxon:** scientificName: *Pancoriuslui* sp. nov.; order: Araneae; family: Salticidae; genus: Pancorius; **Location:** continent: Asian; country: China; countryCode: CHN; stateProvince: Guizhou; county: Shibing; locality: Yuntaishan Scenic Area; verbatimElevation: 900-1000 m; decimalLatitude: 27.13367; decimalLongitude: 108.108833; **Identification:** identifiedBy: Cheng Wang; **Event:** year: 2015; month: 7; day: 30

#### Description

**Figure 1.**
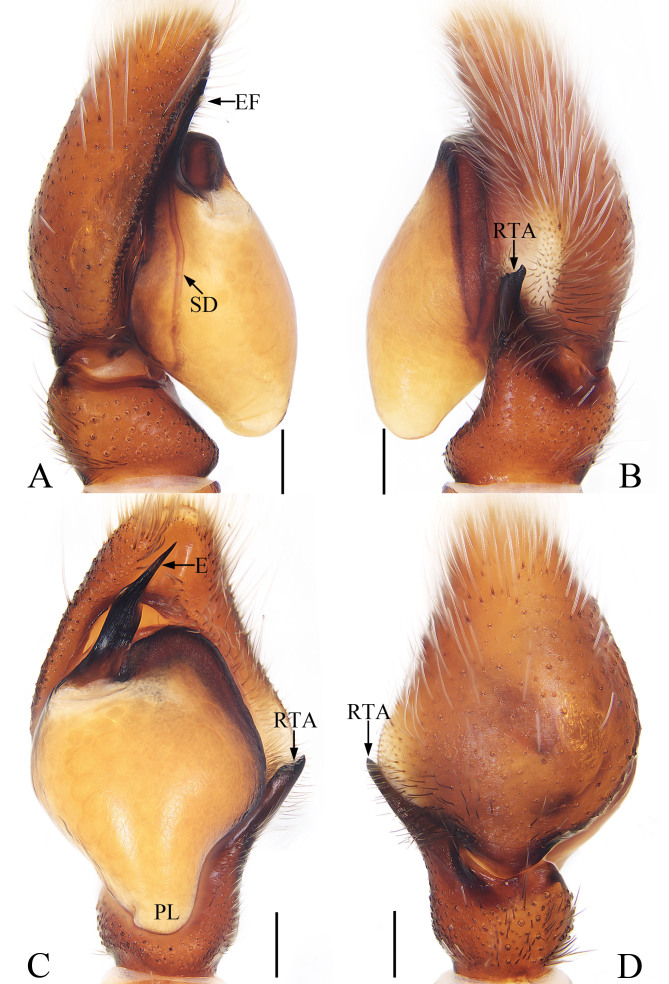
Male palp of *Pancoriuslui* sp. nov., holotype. **A** prolateral; **B** retrolateral; **C** ventral; **D** dorsal. Scale bars: 0.2. Abbreviations：**E** embolus; **EF** embolic flange; **PL** posterior lobe; **RTA** retrolateral tibial apophysis; **SD** sperm duct.

**Figure 2.**
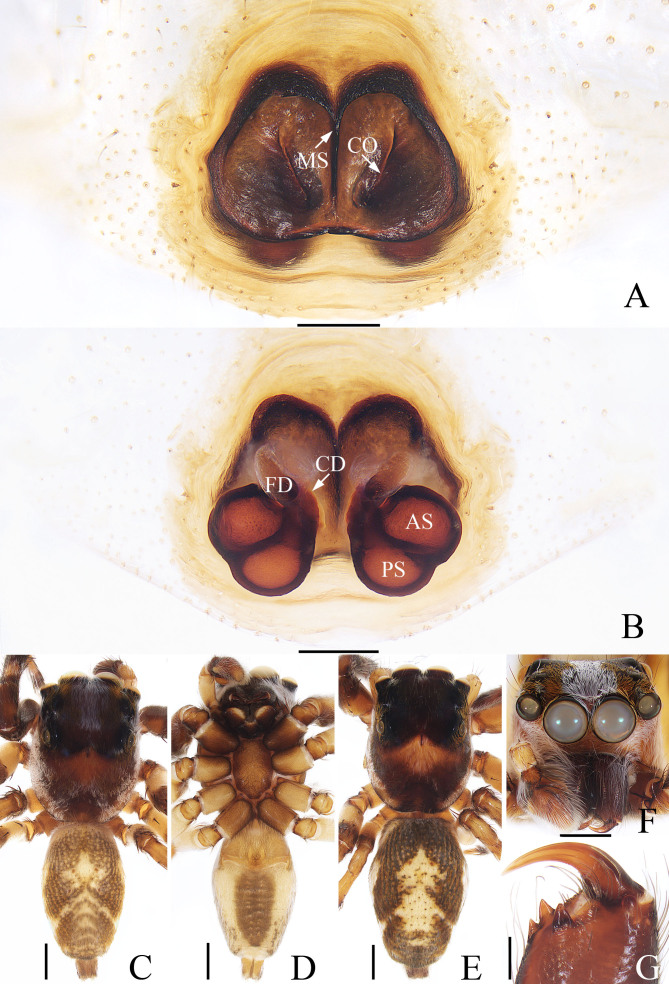
*Pancoriuslui* sp. nov., female paratype and male holotype. **A** epigyne, ventral; **B** vulva, dorsal; **C** male habitus, dorsal; **D** ditto, ventral; **E** female habitus, dorsal; **F** male carapace, frontal; **G** male chelicera, posterior. Scale bars: A, B, G (0.2); C–F (1). Abbreviations: **AS** anterior chamber of spermatheca; **CD** copulatory duct; **CO** copulatory opening; **FD** fertilisation duct; **MS** median septum; **PS** posterior chamber of spermatheca.

Male (Fig. 1, Fig. 2C, D, F and G). Total length 7.89. Carapace 4.01 long, 3.16 wide. Abdomen 3.96 long, 2.36 wide. Eye sizes and interdistances: AME 0.89, ALE 0.53, PLE 0.49, AERW 2.89, PERW 2.71, EFL 1.78. Legs measurements: I 9.93 (3.01, 3.90, 2.01, 1.01), II 9.06 (2.90, 3.50, 1.65, 1.01), III 10.16 (3.30, 3.50, 2.35, 1.01), IV 10.32 (3.20, 3.50, 2.61, 1.01). Carapace red-brown to dark brown, cephalic region bearing a longitudinal, broad, blue-violet band of hairs centrally and a red-brown irregular area anteriorly on thorax, covered with dense, pale and golden hairs. Fovea dark, longitudinal. Chelicerae dark brown, with 2 promarginal and 1 retromarginal teeth. Endites paler than chelicerae, with pale antero-inner margins. Labium somewhat linguiform. Sternum about 1.5 times longer than wide, covered with dark hairs. Legs pale yellow to dark, spinose. Abdomen oval, dorsum brown, mottled, with a pair of longitudinal, marginal stripes and an irregular, anteromedially located yellow patch, followed by three sub-triangular patches and two transverse streaks, covered with dark thin hairs; venter pale yellow laterally, with a broad dark brown patch bearing a pair of dotted lines centrally.

Palp (Fig. 1A–D): tibia short, wider than long; RTA about 1.5 times longer than tibia length, broadened anteromedially, strongly sclerotised at distal half, with two small, triangular sub-apical processes in retrolateral view; bulb swollen, with tapered posterior lobe extending downwards in ventral view; embolus flat, strongly sclerotised, originating from the antero-prolateral portion of bulb, curved medially and tapering at distal half to a pointed tip directed towards about 1 o’clock position, with small, lamellar flange medially.

Female (Fig. 2A, B and E). Total length 9.01. Carapace 4.14 long, 3.29 wide. Abdomen 4.62 long, 3.10 wide. Eye sizes and inter-distances: AME 0.95, ALE 0.55, PLE 0.49, AERW 2.91, PERW 2.81, EFL 1.81. Legs measurements: I 8.55 (2.50, 3.25, 1.95, 0.85), II 7.66 (2.40, 3.01, 1.40, 0.85), III 9.15. (2.85, 3.30, 2.15, 0.85), IV 9.50 (3.00, 3.30, 2.35, 0.85). Habitus (Fig. 2E) similar to that of male, except darker in colour and lacking blue-violet band of hairs centrally on cephalic region.

Epigyne (Fig. 2A and B): wider than long, with large, sub-trapeziform atrium having a narrow median septum; copulatory opening slit-shaped, separated from each other; copulatory duct very short, thick, connected to the posterior chamber of spermatheca; spermatheca divided into two oval chambers; fertilisation duct originating from the antero-inner edge of anterior chamber of spermatheca, anterolaterally extending.

#### Diagnosis

The male of this new species closely resembles that of *Pancoriussubmontanus* Prószyński, 1992 from India and Japan in having similar palpal structure, but differs in the RTA, which is longer than the tibia and acutely narrowed distally in retrolateral view (Fig. 1B) (vs. about half the tibia length and tapered in *P.submontanus*; Prószyński 1992: fig. 117). The female of this species can be distinguished from its congeners by lacking a distinct epigynal hood and having a median septum (see Metzner 2022). The male of this new species also somewhat resembles that of *Evarchalata* Kanesharatnam & Benjamin, 2021 from Sri Lanka in having flat embolus and similarity-shaped RTA, but differs in the short palpal tibia, swollen bulb and the presence of triangular sub-apical processes of RTA (Figs. 1A–D) (vs. much longer palpal tibia, flat bulb and lacking sub-apical process of RTA in *E.lata*; Kanesharatnam and Benjamin 2021: figs. 15e, f and 17c–e).

#### Etymology

The specific epithet is a patronym, after Mr. Qianle Lu (Shenzhen, China) who helped us in collecting numerous specimens of jumping spiders; noun in genitive case.

#### Distribution

**Figure 3.**
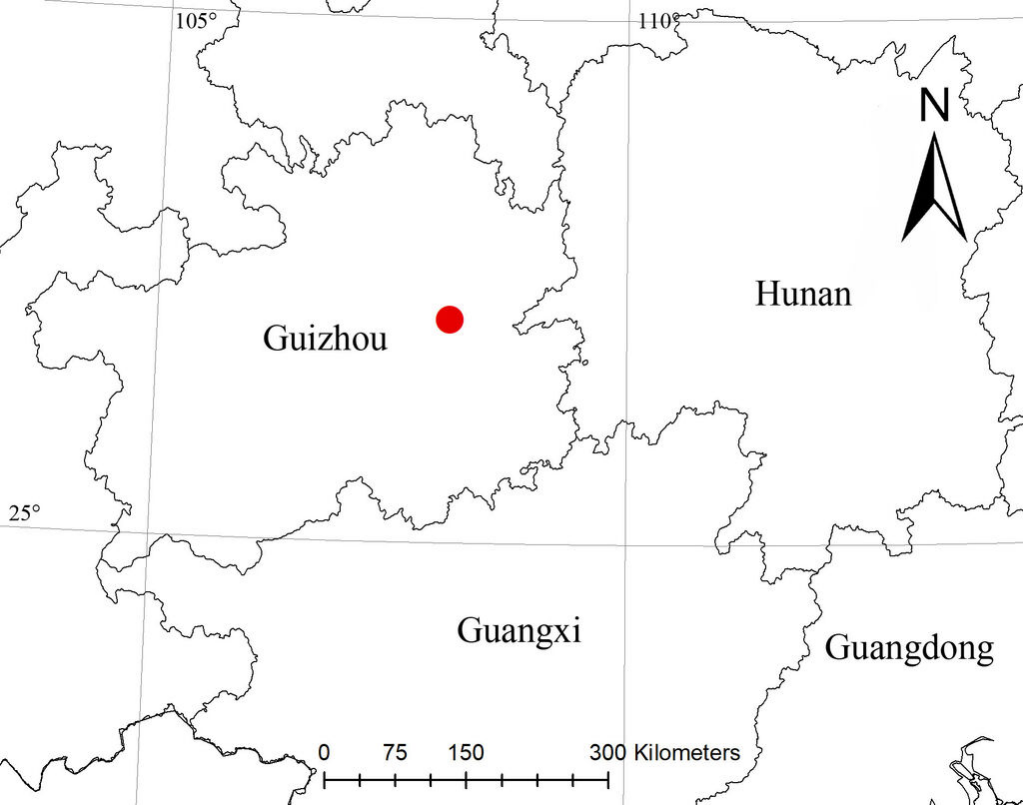
The type locality of *Pancoriuslui* sp. nov.

Known only from the type locality in Guizhou, China (Fig. 3).

#### Taxon discussion

The generic placement is due to the presence of a series of similar features with other *Pancorius* species, such as the band of hairs centrally on eye field, the embolic flange, the slit-shaped copulatory opening and the two-chambered spermatheca. However, this species lacks an epigynal hood and possesses a median septum, which are in contrast with what has been documented in the congeners. Therefore, the generic position of this species may need further confirmation.

## Supplementary Material

XML Treatment for Pancorius lui
